# Body Mass Index Trends for the Top Five Finishers in Men’s Grand Tour and Monument Cycling Events from 1994–2023: Implications for Athletes and Sporting Stakeholders

**DOI:** 10.3390/sports12070178

**Published:** 2024-06-26

**Authors:** Alexander Smith, Helen Wyler, Moritz van Wijnkoop, Jill Colangelo, Michael Liebrenz, Anna Buadze

**Affiliations:** 1 Department of Forensic Psychiatry, University of Bern, Hochschulstrasse 4, 3012 Bern, Switzerlandmoritz.vanwijnkoop@psychologie.ch (M.v.W.);; 2 Faculty of Behavioural Sciences and Psychology, University of Lucerne, 6002 Lucerne, Switzerland; 3 Department of Psychiatry, Psychotherapy and Psychosomatics, Psychiatric Hospital, University of Zurich, 8032 Zurich, Switzerland; ana.buadze@pukzh.ch

**Keywords:** BMI, cycling, weight loss, weight management, performance, eating disorders

## Abstract

Weight-related issues can be prevalent in elite-level sports, especially in men’s road cycling, where riders may exhibit harmful behaviours, with potentially adverse outcomes for mental and physical health. This study investigated Body Mass Index (BMI) values amongst the top five finishers in the three Grand Tours and the five Monuments races between 1994 and 2023 to assess longitudinal patterns. Publicly available height and weight figures were sourced from ProCyclingStats and BMI scores were calculated for *n* = 154 and *n* = 255 individual athletes for the Grand Tours and Monuments, respectively. Two analyses were conducted with correlations and ANOVAs: the first included the BMIs of all top-five finishes and the second focussed on the BMIs of new top-five entrants. The results from both analyses revealed consistent mean BMI decreases over the years and larger effect sizes were apparent in the Grand Tours compared to the Monuments. Although lower BMIs are associated with certain performance advantages, these declining trajectories suggest a need for enhanced awareness in the cycling community and possible regulatory measures and educational programmes to promote the sustainable wellbeing of riders. This may be particularly pertinent given the wider evidence of unhealthy weight-related attitudes and behaviours throughout the sport.

## 1. Introduction

### 1.1. Weight Management Practices, Performance-Based Factors, and Elite-Level Cycling

Weight-loss pressures and unhealthy weight-management patterns in elite-level sports can be detrimental for the physical and mental wellbeing of athletes [1,2]. Across various sporting disciplines, acute weight-loss techniques and pathological behaviours have been identified, including instances of disordered eating, extreme dietary regimes, overtraining, and substance use, e.g., [3,4,5]. These issues may entail risks for clinical and subthreshold eating disorders (EDs), depression, cardiovascular disease, and additional psychiatric and physical conditions [3,4,5,6,7]. The tendency for athletes to adopt harmful weight-management practices can be influenced by socioenvironmental and ergometric considerations, like performance-oriented cultures, body dissatisfaction, extraneous pressures, and competitive advantages linked to body type, weight, and mass [2,5]. The latter may be more pronounced in weight-dependent sports (e.g., gymnastics, rowing, and skiing) where specific anthropometric or physiological characteristics can yield certain advantages for performance [2,5,7,8]. 

Akin to other weight-sensitive disciplines, these dynamics have become increasingly apparent in elite-level cycling due to its competitive demands, environmental determinants, and other factors [9,10,11,12,13,14,15,16,17,18]. Accessible weight and body composition data remain difficult to obtain in this sport, likely due to commercial and cultural considerations, such as the strategic importance for teams in keeping competitor information confidential. Nevertheless, anecdotal and academic evidence suggests cause for concern. Existing reports underline how external pressures and an emphasis on ergometric interactions between body mass, power-to-weight ratios, and performance levels can engender adverse habits as riders seek to attain an “ideal” physique [9]. In this regard, past insights reveal that elite-level cyclists have engaged in harmful activities to maintain or lose weight [6,10,11,12,13]. Resultantly, this has prompted commentators in the cycling community to scrutinise the sport’s “obsession with weight” [10,12,14] and researchers to hypothesise that pathological weight-management practices have become normalised in cycling [11].

Moreover, studies involving elite-level male cyclists illustrate the extent of weight-conscious attitudes across the sport [6,11,15]. Riebl et al. found that male riders had elevated scores on the Eating Attitudes Test-26 compared to the general population, with many participants contending that EDs were somewhat common in cycling [11]. In a sample of men’s elite cyclists from Filaire and colleagues, 46% felt pressured to lose weight [6]. Furthermore, press accounts have described the negative implications of sociocultural paradigms like “eating is cheating”, “win at all costs”, and “fat shaming” [12,16,17]. Equally, cycling support staff often require athletes to follow strict nutritional regimes intra-competition and meet stipulated race weight targets, exacerbating individual weight-related stressors [12,18]. Taken together, these dynamics may serve to heighten the likelihood of harmful weight-management patterns in the peloton, creating concomitant risks for the wellbeing of riders and impacting the wider image of cycling as a health-promoting discipline. 

### 1.2. Governance and Prestigious Competitions in Elite-Level Cycling

As the global governing body for men’s elite-level road cycling, the Union Cycliste Internationale (UCI) manages races and develops regulatory and policy frameworks [19]. Organized by the UCI, the premier division (the UCI WorldTeams) comprises over five hundred male road cyclists in World Tour races [20]. 

The men’s World Tour has three major multi-stage events (the Grand Tours) that are considered the most demanding competitions in the UCI’s calendar; these consist of the Giro d’Italia, the Tour de France, and the Vuelta a España. The Grand Tours offer more points than other World Tour races, which are awarded across several categories, such as the best climbers (Mountains Classification) and fastest sprinters (Points Classification). Of these, the General Classification (GC) charts the cumulative race time of participants and determines overall positions. The Grand Tours are approximately 3500 km in length, with numerous stages involving mountainous terrain, including ~40–60 km of vertical ascent in the Tour de France, ~45–55 km in the Giro, and ~40–50 km in the Vuelta. Accordingly, lower weight has been recurrently acknowledged as a beneficial trait for competing riders (e.g., [21,22,23,24]). 

Alongside the Grand Tours, the UCI oversees five prestigious Monuments races: the Milan–San Remo, the Tour of Flanders, Paris–Roubaix, Liège–Bastogne–Liège, and the Giro di Lombardia. Unlike the multi-stage Grand Tours, the Monuments are single-day events with only GC standings and typically encompass flatter course designs, sprinting segments, and lower elevation gain [25]. For instance, Milan–San Remo is known for its length (~298 km) and sprint finish, whilst Paris–Roubaix has challenging cobblestone sections. Consequently, weight may be less of a significant performance determinant in certain Monuments and instead, these races may require a greater emphasis on tactical acumen and sprinting skills over shorter intervals. 

### 1.3. The Current Study

With the associations between performance and weight in men’s elite-level road cycling, alongside evidence about socioenvironmental weight-loss pressures and unhealthy practices amongst riders, this study aimed to investigate wider patterns across the sport. Specifically, in lieu of systematically accessible and longitudinal weight data, we sought to examine Body Mass Index (BMI) scores from the top five finishers in Grand Tour and Monument events between 1994–2023. 

Albeit a disputed indicator [26], BMIs are extensively utilised for measuring weight adjusted for height (kg/m^2^), notably by the World Health Organization (WHO), which defines <18.5 kg/m^2^ as being underweight and 18.50–24.99 kg/m^2^ as normal [27]. Similarly, in psychiatric practice, BMIs are embedded in the Diagnostic and Statistical Manual of Mental Disorders (DSM) as markers for low weight (i.e., mild: BMI ≥ 17 kg/m^2^, moderate: BMI 16–16.99 kg/m^2^, severe: BMI 15–15.99 kg/m^2^, and extreme: BMI < 15 kg/m^2^) [28]. Using BMIs as a parameter to examine weight-related issues in sport has limitations, as this metric does not differentiate between body types and can be shaped by different factors; specifically, in cycling, distinct anthropometric characteristics and body compositions can influence power output and competitive performance [29,30,31]. Nevertheless, together with other determinants, low BMIs can have associations with adverse psychiatric and somatic health outcomes (e.g., [27,28,32,33,34]), which could be potentially detrimental to the short- and long-term wellbeing of athletes.

Our primary hypothesis assumed that we would observe declining BMI metrics for the top finishers in the Grand Tours and the Monuments from 1994–2023, owing to an increased emphasis on body composition within cycling and the sport-specific socioenvironmental pressures discussed above [9,10,11,12,13,14,15,16,17,18]. If discernible, such trends could demonstrate a need for greater awareness and considerations about possible regulatory interventions to promote the health and wellbeing of riders in both the short and long term. Moreover, as a secondary hypothesis, we predicted there would be more significant decreases in BMI trajectories in the Grand Tours than the Monuments. This was based on the multi-stage nature and higher elevation of the Grand Tours, where lower BMIs may confer greater competitive advantages [21,22,23]. 

## 2. Methods

### 2.1. Design

This study employed a retrospective longitudinal design to examine secondary, publicly available data on the top five GC classifications of elite-level male cyclists in the three Grand Tours and the five Monuments races between 1994 and 2023. The objective of this research was to evaluate BMI trends amongst these riders over this timeframe. 

### 2.2. Procedure

**Figure 1:**
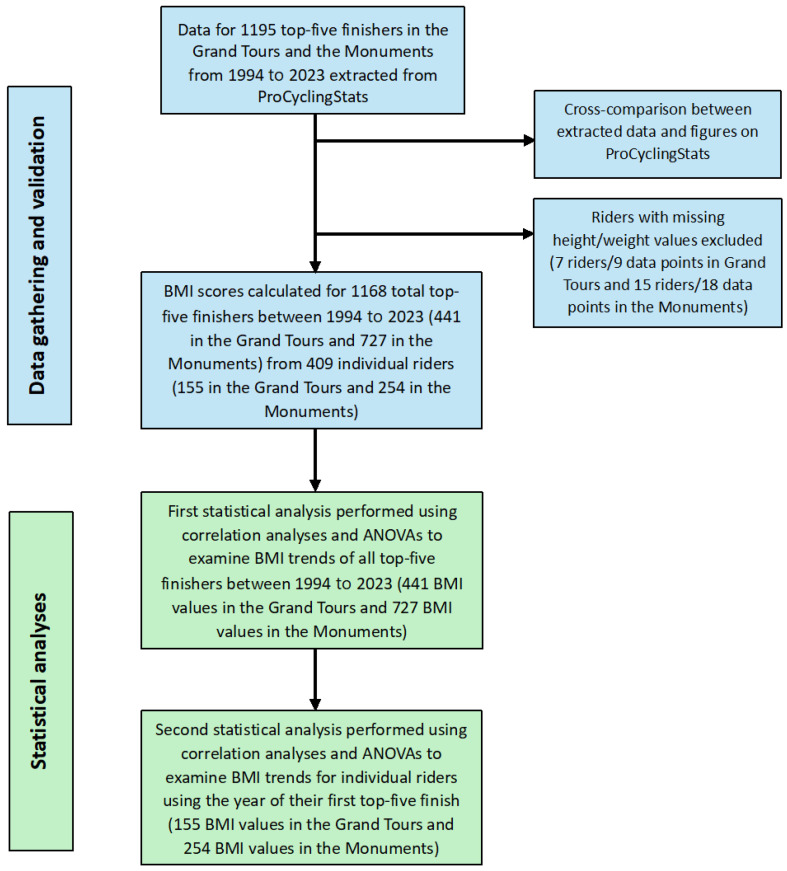
Flowchart of methodological procedures in this study.

The methodological procedure for obtaining and analysing the BMI data in this study is outlined in Figure 1.

#### 2.2.1. Data Collection and Validation

Due to the scarcity of accessible anthropometric data formally and systematically disclosed by teams or riders, we collected information from the website ProCyclingStats [35]. This website displays details on elite-level cycling from various sources, including individual riders, teams, and community contributors (email communication ProCyclingStats, October 2022). In March 2024, one member of the research team gathered data from ProCyclingStats for each top five finisher in every Grand Tour and Monuments race from 1994–2023 (note that the 2020 edition of the Paris–Roubaix was cancelled due to COVID-19 restrictions). This process entailed manually collecting publicly available information about the cyclist’s name, weight (kg), height (cm), year of birth, first year of top five entry, and race year from ProCyclingStats. Subsequently, two members of the research team cross-referenced these extracted data with the values displayed on ProCyclingStats to uphold accuracy and verify collated figures.

Based on the height and weight data from ProCyclingStats, BMI values were calculated for each cyclist. Riders could achieve multiple top five positions over the duration of the observation period and also across different races within the same year. Thus, the BMI of an individual competitor may be represented more than once in the overall dataset (see Section 2.2.3 for how this was addressed). Equally, it should be noted that ProCyclingStats exhibits a single height and weight metric for each rider, regardless of the year and stage of the season. Therefore, it was not possible to account for annual changes in an individual cyclist’s BMI or BMI changes within a given season. 

#### 2.2.2. Sample Description

450 datapoints were available in the three Grand Tours. For seven athletes, no height or weight information was shown on ProCyclingStats and these were excluded from the analysis (amounting to nine datapoints, as some riders achieved more than one top five GC finish). Accordingly, this resulted in a total of 441 datapoints for the top five GC standings in the Grand Tours from 1994–2023. Within this, the dataset included 155 individual riders with at least one top five classification, for whom all information was available from ProCyclingStats. 

For the Monuments, a total of 745 datapoints were calculated from 1994–2023. For 15 riders, no weight or height information was available from ProCyclingStats and, again, these were excluded from the analysis (amounting to 18 datapoints as some riders achieved more than one top-five position). This resulted in a total of 727 datapoints over the thirty-year period for riders with top-five finishes in the Monuments. Across these events, there were 254 individual riders with at least one top five GC, for whom all information was presented by ProCyclingStats. 

#### 2.2.3. Data Analyses

Data analyses were performed using SPSS 29 (IBM, Chicago, IL, USA). An alpha of 0.05 was set and two-tailed tests are reported. Confidence intervals for correlations were based on bootstrapping with 1000 samples.

We conducted the main analyses using two approaches. In the overall analysis, we included the top five athletes for whom the relevant data were available for each year and each race. Hence, individuals who achieved top-five positions repeatedly carry more statistical weight in these analyses. To check whether the results were affected by including repeated data and to assess BMI changes amongst new top five entrants, we performed a second analysis. Within this, each rider was included only once, specifically for the year of their first entry into a top-five position.

Correlation analyses were performed to examine the association between year of a top-five standing (or year of first top-five standing) and BMI. One-way ANOVAs were calculated to investigate how the BMI values of the riders compare across the three decades included in the analyses (1994–2003; 2004–2013; 2014–2023). Effect sizes are reported with small, medium, and large effect sizes corresponding to 0.10, 0.30., and 0.50 for Pearson’s *r*, 0.20, 0.50, and 0.80 for Cohen’s *d*, and 0.01, 0.06, and 0.14 for η^2^_p_ [36].

### 2.3. Ethical Considerations 

This study used publicly available secondary data from ProCyclingStats and did not involve any direct participation from cyclists or human subjects. Nevertheless, owing to potential sensitivities surrounding BMI and weight data, ethical approval was sought from the Internal Review Board (IRB) of the Faculty of Philosophy and Human Sciences Ethics Commission at the University of Bern. On 11 January 2023, the methodology was approved by this Commission as deemed to entail no ethical harms. 

## 3. Results

### 3.1. Descriptives

Table 1 provides an overview of descriptive information for the elite-level male cyclists included in the Grand Tour sample (*n* = 162 for age at first top-five GC position and number of top-five classifications, all other *n* = 155) and the Monuments sample (*n* = 269 for first top-five GC position, all other *n* = 254). Top five finishers in the Grand Tour were both lighter (medium to large effect) and shorter (small effect) than those in the Monuments, and they also had a lower BMI, with a medium-to-large effect size. There were no statistically significant differences regarding the number of top five classifications or the age at the first top five classification of a rider between the Grand Tours and the Monuments. 

### 3.2. Grand Tour Races

**Figure 2:**
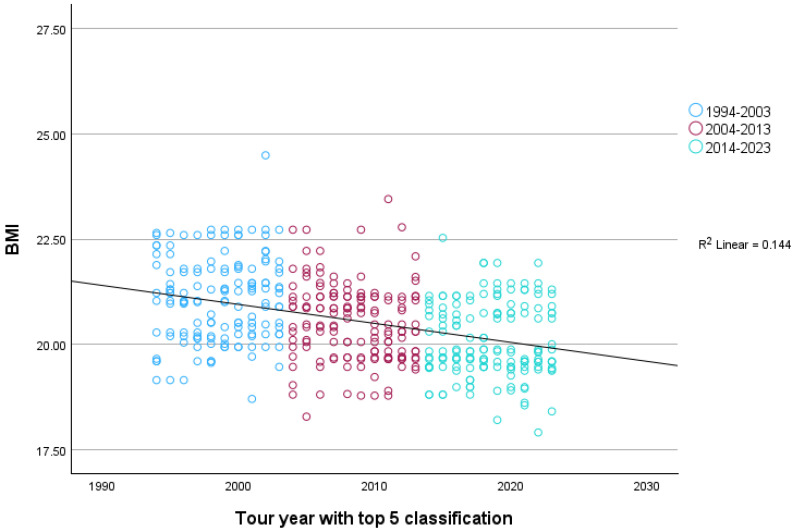
BMI trends of all top five finishers in the Grand Tours from 1994–2023.

As displayed in Figure 2, for the Grand Tours, there was a statistically significant association between the tour year with a top-five classification and the BMI of riders, *r* (*n* = 441) = −0.38, 95% CI [−0.46, −0.30], *p* < 0.001, with the result corresponding to a medium to large effect. This remained very similar if each cyclist was included only once at the year of their first entry into a top-five position, *r* (*n* = 155) = −0.39, 95% CI [−0.50, −0.24] *p* < 0.001. 

When comparing the three decades (1994–2003; 2004–2013; 2014–2023), a one-way ANOVA revealed a main effect of tour year of a top-five position, *F* (2, 438) = 40.10, *p* < 0.001, η^2^_p_ = 0.16, 95% CI [0.10, 0.21]. The observed effect was large. Post-hoc tests showed that there was a statistically significant decrease in average BMI from each decade to the next (1994–2003: *M* = 21.08, *SD* = 1.01; 2004–2013: *M* = 20.53, *SD* = 0.91; 2014–2023: *M* = 20.10, *SD* = 1.02), all *p*s < 0.001. 

**Figure 3:**
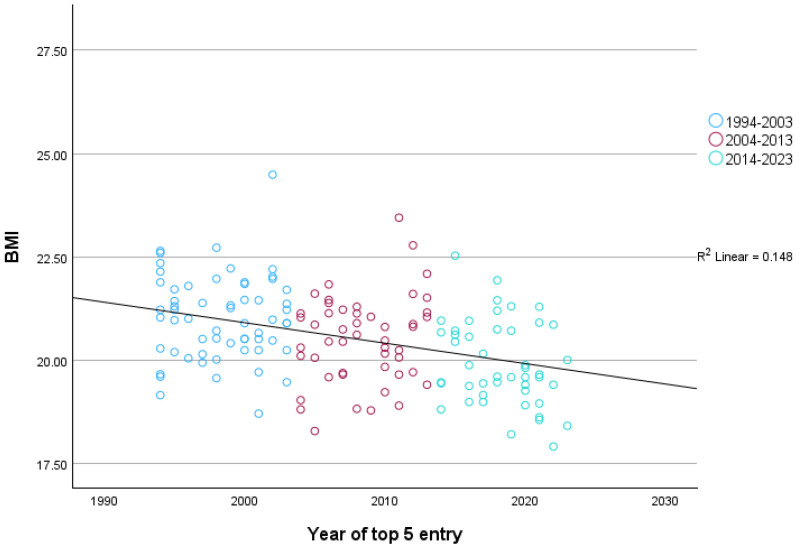
BMI trends for top five finishers in the Grand Tours, including only their first top-five entry.

Comparable results were obtained when only including the first top-five finish for each rider, *F* (2, 152) = 15.87, *p* < 0.001, η^2^_p_ = 0.16, 95% CI [0.06, 0.26], as highlighted in Figure 3. Post-hoc comparisons revealed that the three decades were all statistically significantly different from each other (all *p*s < 0.034). In the first decade, the average BMI was 21.03 (*SD* = 1.02), in the second decade this dropped to 20.52 (*SD* = 1.06), and in the third it reduced further to 19.92 (*SD* = 1.03).

### 3.3. Monuments

**Figure 4:**
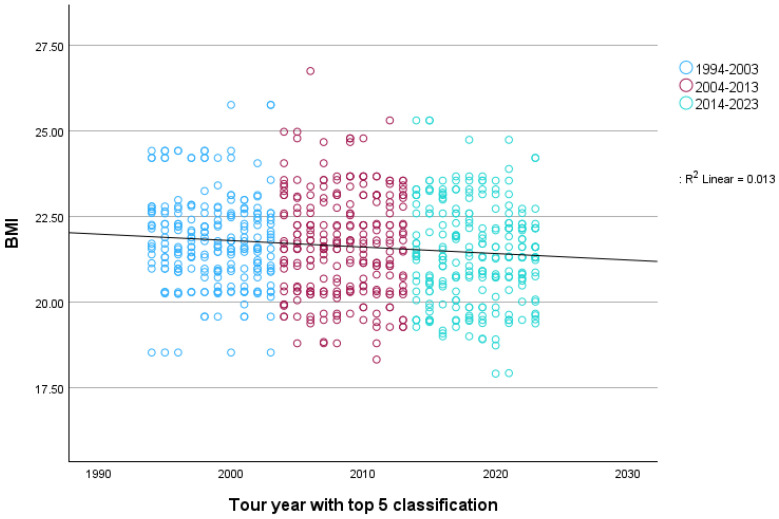
BMI trends of all top five finishers in the Monuments from 1994–2023.

**Figure 5:**
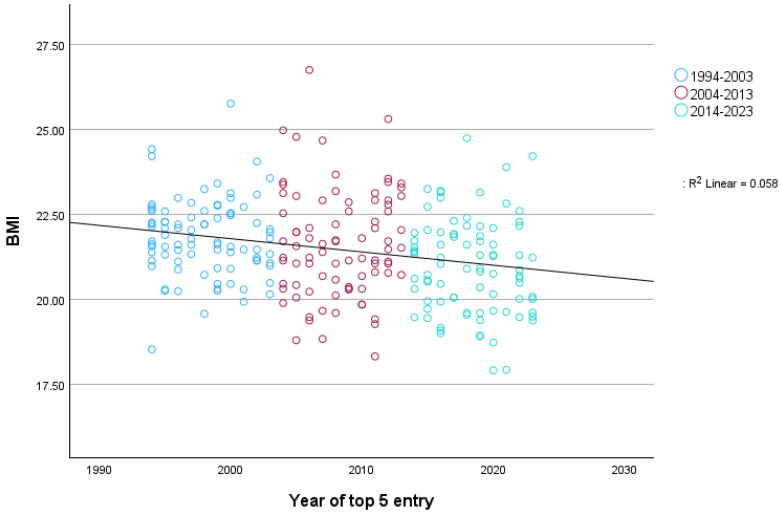
BMI trends for top five finishers in the Monuments including only their first top-five entry.

For the Monuments, as presented in Figure 4, there was a statistically significant association between race year with a top-five classification and BMI, *r* (*n* = 727) = −0.11, 95% CI [−0.18, −0.04], *p* = 0.002, although the observed effect was small. If each rider was included only once in the year of their first entry into a top-five classification, the association was *r* (*n* = 254) = −0.24, 95% CI [−0.35, −0.13], *p* < 0.001, which corresponds to a small-to-medium effect, as represented in Figure 5.

When comparing the three decades (1994–2003; 2004–2013; 2014–2023), the assumption of Levene’s test of homogeneity of variance was violated. The results of a one-way Welch’s ANOVA revealed that the main effect was tour year of a top-five classification, *F*(2, 482.66) = 4.17, *p* = 0.016, η^2^_p_ = 0.01, 95% CI [0.00, 0.03], which corresponded to a small effect. Post-hoc comparisons showed a statistically significant difference between 1994–2003 (*M* = 21.79, *SD* = 1.32) and 2014–2023 (*M* = 21.43; *SD* = 1.45), *p* = 0.013, whereas 2004–2013 (*M* = 21.68; *SD* = 1.49) did not differ statistically significantly from either of the other two decades, *p*s > 0.136.

Equally, the assumption of homogeneity of variances was violated when only including the first top-five classification for each athlete. Comparable results were observed, with a one-way Welch’s ANOVA suggesting a medium effect, *F* (2, 163.11) = 9.74, *p* < 0.001, η^2^_p_ = 0.07, 95% CI [0.02, 0.13]. Post-hoc comparisons revealed that whilst there was no statistically significant difference between the first two decades (*p* = 0.599), the average BMI was significantly lower in the most recent decade (*M* = 20.96, *SD* = 1.41) compared to the previous two (1994–2003: *M* = 21.82, *SD* = 1.14, *p* < 0.001; 2004–2013: *M* = 21.61, *SD* = 1.59, *p* = 0.015).

## 4. Discussion

### 4.1. BMIs and Sporting Performance in the Grand Tours and the Monuments

These analyses show statistically significant decreases in BMI trends for the top five men’s road cyclists in the GC of the Grand Tours between 1994–2023, identifying a large effect size within decade-by-decade analyses. To a lesser extent, declining BMI trajectories were also apparent for the top five finishers in the Monuments races during this thirty-year period, especially for 2014–2023. Consequently, these findings support our primary hypothesis predicting generalised patterns of decreasing BMIs for the top five finishers across all included races over time, alongside our secondary hypothesis that such trends would be more pronounced in the Grand Tours than the Monuments. 

For the Grand Tours, the results corroborate prior scholarly work, indicating that lower BMIs may be increasingly associated with top GC finishing positions [21,22,23]. For example, using a model based on the 2004 Tour de France, Torgler showed that lower BMIs substantially increased the probability of finishing in the final top twenty-five positions [21]. Likewise, Prinz and Wicker evaluated Tour de France performances between 2002 and 2004 and found that lower BMIs had a positive impact on the ranking of individual riders [22]. Additionally, Coupe and Gergaud reported that higher BMIs negatively influenced performance in the Tour de France as measured by cumulative times and finishing positions [23]. Elsewhere, researchers have discussed that weight and body mass can be beneficial to cyclists seeking to attain competitive advantages in mountain terrain and during intervals of elevation gain [37,38]. 

It should be noted that BMI may not always serve as a reliable indicator of an athlete’s physical condition and this metric can be changed by different variables in sporting contexts over a relatively short period of time (e.g., dehydration). Likewise, BMIs do not account for variations in muscle or fat percentage in athletes, which are factors that can also affect cycling performance [29,30,31,38]. That said, competitive dynamics may help to explain the varying declines in BMI trajectories between the Grand Tours and the Monuments. Notably, as multi-stage competitions, Grand Tour courses typically feature numerous sections with substantial vertical gain and mountain climbing, thereby favouring riders who have power curves with sustained high-power outputs; for instance, the 2024 Tour de France course alone has a total elevation gain of 52,230 m [39]. Nevertheless, for athletes seeking to achieve success in alternative Grand Tour categories, like sprinters in the Points Classification, maintaining a lower body mass could be deemed less advantageous because of the importance of peak power curves over shorter distances and flatter sections [37,38]. In this regard, with their comparatively higher body weight, sprinters are often at the back of the peloton during the Grand Tour in the so-called “grupetto” [40], which consists of riders who struggle with climbing and work together to finish within the time limit to avoid exclusion and ensure they remain in the competitions for flatter stages. 

These may be equally apposite considerations in the Monuments events, which are single-day races and tend to encompass fewer cumulative vertical climbing sections and more sprinting. In Monuments courses with less elevation, riders with a higher (maximum) absolute power in watts tend to surpass those classified as climbers, allowing them to maintain a higher pace on undulating terrain, effectively flattening shorter climbs. Therefore, in the authors’ opinion, it could follow that relatively higher BMIs in certain Monument events could be linked to optimised power output and competitive success. However, again, physiological interconnections between body mass, body composition, power, and ergometry are multifaceted in elite-level cycling [29,30,31,37,38], potentially confounding correlations between BMI and performance, and specific Monuments can vary in terms of course designs and racing demands. 

### 4.2. BMI Trends and Health Implications

Regardless of the inter-race nuances across Grand Tours and Monuments, our results showed year-by-year decreases in BMI values for the top five finishers in all competitions. This was especially notable in 2013–2023 compared to prior decades. The mean BMI values for these events did not meet indicative threshold markers from the WHO or the DSM for weight-related issues [27,28]. That said, the longitudinal downward trajectories should raise concerns for the cycling community, particularly if current trends continue, as they could affect the athletic capacity and overall wellbeing of riders. 

This is a relevant consideration given the potential performance advantages of lower BMIs for riders, alongside other socioenvironmental determinants, which may encourage harmful weight-management techniques previously outlined within the sport [9,10,11,12,13,14,15,16,17,18]. This includes fasting, self-induced vomiting, extreme dieting, overtraining, laxative use, and weight cycling in the lead-up to races [6,9,10,11,12,13]. In the general population, the literature indicates that low BMIs and extreme weight-loss practices can constitute risk factors for various physical morbidities and mental health disorders (e.g., [32,33,34,41,42,43]). Specifically, persistently low BMIs, low weight, and reduced body fat in athletes could have associations with compromised physiological functioning and psychiatric vulnerabilities (e.g., [44]). Separately, there have been ongoing discussions about detrimental outcomes resulting from low energy availability in athletes and relative energy deficiency in sport (REDs) [45]; the latter has been linked to osteoporosis, poor immune function, and impaired performance [46]. Male cyclists have been found to be vulnerable for REDs, which may continue to impact their health into retirement [47]. Likewise, a sizeable incidence of low bone mineral density and higher vulnerabilities for bone fractures have also been identified amongst elite-level riders [48,49]. Additionally, in prior situations, weight-related pressures have resulted in medicolegal sanctions for elite-level cyclists, with one competitor receiving a doping sanction for consuming a prohibited dietary supplement [17]. 

Beyond elite-level domains, detrimental eating behaviours have been highlighted in junior competitions [10]. Moreover, commentators have described “copycat” behaviours among amateur participants, who may associate low weight with sporting success and seek to emulate the body type of top-level riders [10,14]. Related findings were shown in a past study incorporating well-trained amateur cyclists, where a substantial proportion of the sample affirmed that they had recently tried to or were currently attempting to lose weight [50]. Notably, when asked to provide advice to those “starting in the sport”, a key recommendation from a prominent elite-level male rider was that “a very important thing is to be light” [51]. Conceivably, these aesthetic and cultural influences could amplify the “obsession with weight” across cycling and influence wider attitudes towards attaining specific BMIs [10,12,14]. As implied by past press coverage [52], images of elite-level riders exhibiting unhealthy weight profiles may undermine the connotations of cycling as offering “health benefits”, which is an institutional goal of the UCI [53].

### 4.3. Considerations for Sporting Stakeholders in Elite-Level Cycling

When interpreted within this wider context, the consistent decline in BMI trajectories in our findings, alongside accounts of weight-related issues [9,10,11,12,13,14,15,16,17,18], may underline a need for greater attention from sporting stakeholders and regulators to address potentially unhealthy patterns in cycling competitions. In different weight-sensitive sports, governing bodies have intervened to attenuate these issues by introducing measures based on mass or weight. This includes the International Ski and Snowboard Federation (ISF), who in 2004 specified that ski length must be predicated on the BMI scores of participants [54]. In limiting competitive advantages by BMI-based criteria, the ISF’s regulations have been effective in countering EDs and problems related to low weight, although they have been criticised for excluding outlying body compositions [54]. Correspondingly, in 2019, the Fédération Internationale de l’Automobile amended the car and driver weight calculation in Formula One, stipulating the lowest driver mass. As of the 2024 season, the minimum weight for drivers including their helmet and head and neck support is 80 kg and drivers must wear race gear when they are being weighed [55]. Should a driver’s weight and equipment be less than 80 kg, ballast must be added to the cockpit area in compliance with FIA weight limits. 

These precedents demonstrate how regulatory bodies in other sports have successfully enacted measures to address weight-related issues, in turn aiming to promote healthier practices. In elite-level cycling, more transparency about weight data and active stakeholder engagement would be required to inform considerations about possible and significant regulatory changes. Nevertheless, basic measures could be implemented to improve awareness about the health consequences of low BMI and the performance-oriented culture that pervades the sport. To that end, the UCI could consider compulsory regular weight and body composition screenings for riders and obligatory follow-up measures for athletes outside of a healthy range. These may be particularly applicable intra-race, since riders can seek to drop weight prior to these events [13], and would complement broader UCI recommendations about mental health screening provisions in cycling [56].

Furthermore, psychoeducation initiatives could allow riders to better recognise risk factors [57], underpinned by culturally tailored resources that convey the short- and long-term dangers of harmful weight loss practices (e.g., weight cycling). Similarly, increasing access to evidence-based dietary materials and counselling would be important for competitors, as limited nutritional knowledge has been linked to the development of EDs in riders [58]. Detailed training would also be useful for support teams, including coaches and medical and nutritional staff. In other sports, research demonstrates that elite-level coaches lack sufficient education and have difficulty distinguishing pathological behaviours, thereby underscoring a necessity for comprehensive education schemes [59].

## 5. Limitations and Directions for Future Research

Our analysis of secondary BMI data from the Grand Tours and the Monuments adds to ongoing dialogues around the relationship between low weight and performance benefits in elite-level cycling, which could have health consequences for athletes. Nonetheless, the adopted methodology has several limitations. Firstly, the validity of BMIs has attracted criticism in sports medicine literature, since this metric measures ponderosity rather than fat and does not account for outlying body types, which could be a salient consideration in elite-level cycling [29,60]. However, in lieu of systematically accessible weight and body composition data, we deemed BMIs to be an appropriate indicator to gain perspectives about broader patterns in this sport, and they are commonly utilised in clinical practice (e.g., [26,27,28]). 

Moreover, we only investigated male road cyclists in the GC of the Grand Tours and the Monuments, which limits the applicability of our findings to a specific subset of competitors. As a direction for additional research, different UCI events or classifications could be examined to ascertain wider trajectories. Likewise, we solely focussed on men’s races, as comparative data for female events were not available. Two women’s WorldTour stage races have been established recently (the Tour de Frances Femmes in 2022 and Challenge by La Vuelta in 2020), thereby negating the possibility of conducting longitudinal analysis. As there are gender disparities in the exhibition of EDs and related research [2], and adverse weight-management behaviours have been identified in women’s cycling [9,57,61], it would be important to chart historical BMI trends in the Giro d’Italia Femminile; this has been running consecutively from 1988, although the 2021 edition lost UCI WorldTour status. Correspondingly, detailed investigations involving female cyclists with both low BMI and low body fat content could offer valuable data about weight-related concerns in women’s cycling, as these factors could have sizeable health consequences for these riders [9,57,61]. 

Using ProCyclingStats as a source may raise concerns about data reliability since information on the site is provided by individual riders, teams, and community contributions (email communication, October 2022). The dataset was based on a rider’s GC standing at the time of the race and athletes who were subsequently disqualified for doping transgressions were not removed from the dataset. This approach aimed to ensure consistency and comprehensiveness by accounting for undetected doping violations. Additionally, ProCyclingStats displays a single weight and height point for each rider. Therefore, it may not accurately capture contemporaneous data or changes over short or long periods of time. Likewise, should a rider achieve top-five positions repeatedly over the years, this could skew the results. Hence, to account for possible biases in the results, we conducted one analysis including all top five finishes and a separate assessment where each rider was included only once upon their first top-five entry. 

Our findings should be interpreted with these limitations in mind and provide preliminary insights into these issues. However, previous inquiries into elite-level cycling have used ProCyclingStats as their data source, e.g., [62,63]. Thus, we deemed our methodology to be suitable for assessing larger, longitudinal trends. We would welcome greater transparency around weight data from teams to provide more detailed evaluations and enhance specific evidence-based recommendations. Equally, to mitigate the limitations of solely using BMIs in future research on weight-related issues in cycling, considering body composition analysis and other metrics like Lean Mass Index could yield more comprehensive data about riders’ health and possible sport-specific risk factors [64].

## 6. Conclusions

We calculated secondary BMI data for the top five cyclists in the UCI’s Grand Tours and Monuments between 1994–2023. Our findings indicated a longitudinal decrease in BMI trajectories amongst the top finishers across these races, with more discernible trends in the Grand Tours compared to the Monuments. In the context of wider evidence showing harmful weight-loss approaches and socioenvironmental pressures in elite-level cycling, the consistent decrease in BMI values underscores a possible need for increased attention from sporting stakeholders. 

Though lower BMIs may be associated with competitive advantages, particularly in races involving mountainous terrain, the potential for mental and physical health consequences warrants careful monitoring. Therefore, future regulatory measures could be considered and further research will be needed to inform the feasibility and success of these initiatives. Regardless, we believe that the cycling community must recognise the short- and long-term risks of adverse weight trends and balance performance demands with the sustainable wellbeing of riders on and off the bike. 

## Figures and Tables

**Table 1:** Descriptive information on male elite-level cyclists in the two samples.

	Grand Tour			Monuments			
	*M*	*SD*	Min–Max	*M*	*SD*	Min–Max	Cohen’s *d*
Weight (kg)	64.66	5.00	48–79	69.04	6.81	53–94	−0.71 ***
Height (cm)	177.39	5.80	162–191	179.23	6.33	164–199	−0.30 **
BMI	20.54	1.12	17.92–24.49	21.46	1.43	17.90–26.75	−0.70 ***
Age at first top-five GC	27.37	3.27	20–41	27.54	3.29	21–38	−0.05 ***
Number of top-five classifications	2.78	2.59	1–14	2.77	2.91	1–17	−0.00 ***

Note. Cohen’s *d* describes the effect size of the comparisons between the two groups. *** Difference statistically significant at *p* < 0.001; ** Difference statistically significant at *p* < 0.01.

## Data Availability

All data generated or analyzed during this study is included in the article as Table(s) and Figure(s).
